# Contrasted transmission efficiency of Zika virus strains by mosquito species *Aedes aegypti, Aedes albopictus* and *Culex quinquefasciatus* from Reunion Island

**DOI:** 10.1186/s13071-020-04267-z

**Published:** 2020-08-06

**Authors:** Yann Gomard, Cyrille Lebon, Patrick Mavingui, Célestine M. Atyame

**Affiliations:** UMR PIMIT (Processus Infectieux en Milieu Insulaire Tropical), Université de La Réunion, INSERM 1187, CNRS 9192, IRD 249, Plateforme Technologique CYROI, Sainte-Clotilde, La Réunion France

**Keywords:** *Flavivirus*, Mosquito vectors, Vector competence, Reunion Island

## Abstract

**Background:**

Zika virus (ZIKV) is a mosquito-borne flavivirus that recently emerged in the South Pacific islands and Americas where unprecedented outbreaks were reported. Although *Aedes aegypti* is considered to be the main vector for ZIKV, other mosquito species have been shown to be potential vectors and differences in vector competence with respect to mosquito strain and ZIKV strain have been demonstrated. In this study we compared the vector competence of three mosquito species *Aedes albopictus*, *Ae. aegypti* and *Culex quinquefasciatus* from Reunion Island for three ZIKV strains.

**Methods:**

Five mosquito strains (2 strains of *Ae. albopictus*, 1 of *Ae. aegypti* and 2 of *Cx. quinquefasciatus*) were exposed to three ZIKV strains: one African strain (Dak84) and two Asian strains (PaRi_2015 and MAS66). The vector competence parameters (infection rate, dissemination efficiency and transmission efficiency) and viral loads were examined at 14 and 21 days post-infection.

**Results:**

The two *Cx. quinquefasciatus* strains did not become infected and were therefore unable to either disseminate or transmit any of the three ZIKV strains. *Aedes albopictus* and *Ae. aegypti* strains were poorly competent for the two Asian ZIKV strains, while both mosquito species displayed higher infection rates, dissemination and transmission efficiencies for the African ZIKV Dak84 strain. However, this African ZIKV strain was better transmitted by *Ae. aegypti* as compared to *Ae. albopictus*.

**Conclusions:**

Our results show that both *Ae. albopictus* and *Ae. aegypti*, from Reunion Island, are more likely to be competent for ZIKV in contrast to *Cx. quinquefasciatus* which appeared refractory to all tested ZIKV strains. This improves our understanding of the role of mosquito species in the risk of the ZIKV emergence on Reunion Island.
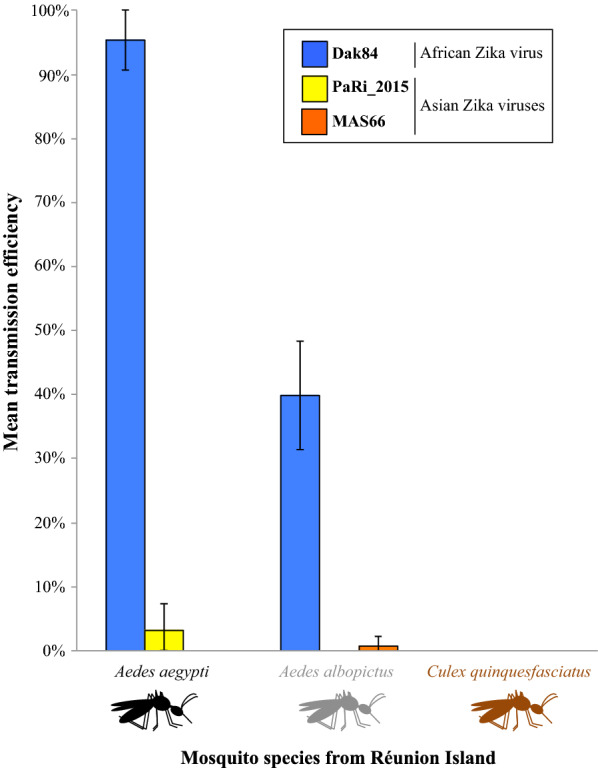

## Background

During the last decades, several mosquito-borne viruses belonging to the families *Togaviridae*, *Phenuiviridae* and *Flaviviridae* have (re)emerged around the world with consequences for the health of human and animal populations [1]. This is, for example, the case of the Zika virus (ZIKV), an emerging flavivirus (family *Flaviviridae*), responsible for large outbreaks in human populations in many countries [2]. Zika virus was first isolated in 1947 from a sentinel rhesus monkey in the Zika Forest (Uganda) and then from *Aedes africanus* mosquitoes in 1948 [3]. Before its global expansion, this virus has received little attention and remained less studied. In 2007, the first ZIKV outbreak was reported in the Yap Islands (Federated States of Micronesia) where 7381 inhabitants (73% of the population) were estimated to be infected [4]. Thereafter, in 2013–2015 a large ZIKV outbreak was reported in French Polynesia, where about 28,000 human cases were recorded [5]. In 2015, the virus reached Brazil and rapidly spread across the Americas where hundreds of thousands of individuals were infected [6]. In humans, symptomatic ZIKV infections are self-limiting acute febrile disease with symptoms ranging from rash, fever, headache and myalgia [7–9]. However, more severe symptoms including microcephaly in newborns and young children and Guillain-Barré Syndrome may occur [6, 10]. Genetic analyses have shown that global ZIKV strains belong to three genotypes; Asian, West African (Nigerian cluster) and East African (MR766 prototype cluster) [11]. The viral strains involved in all major international ZIKV outbreaks belong to the Asian genotype [11–14].

ZIKV has been detected in field isolates of many mosquito genera including *Anopheles*, *Aedes*, *Culex*, *Eretmapodites* and *Mansonia*, and vector competence experiments in laboratory conditions have been performed using mosquitoes from the genera *Anopheles*, *Aedes* and *Culex* [15, 16]. According to these studies, *Aedes aegypti* is considered to be the main vector of ZIKV [16] but other *Aedes* species such as *Aedes albopictus* or *Aedes polynesiensis* also play a role in ZIKV transmission [17, 18]. Conversely, many studies showed that the mosquito species *Culex pipiens* and *Culex quinquefasciatus* play no role in ZIKV transmission [19–29]. As reported for other arboviruses (see [30] for review), the vector competence for ZIKV varies between mosquito strains and ZIKV genotypes [31–34]. For instance, Aubry et al. [35] showed that African *Ae. aegypti* are less susceptible than non-African *Ae. aegypti* to Asian and African ZIKV infections. Similarly, the vector competence of *Ae. albopictus* for ZIKV strains also depends on the geographical origin of field-derived mosquito populations [33, 36]. Therefore, to better evaluate the role of mosquito vectors in the transmission dynamics of arboviruses in the field, it is essential to integrate different genetic variants of both mosquitoes and viruses in the examination of vector competence.

Reunion Island is an overseas French territory located in the South-Western Indian Ocean (SWIO), 700 km East of Madagascar. Twelve mosquito species (belonging to four genera: *Aedes*, *Anopheles*, *Culex* and *Orthopodomyia*) are currently recognized on the island [37] but *Ae. albopictus* and *Cx. quinquefasciatus* are the most abundant mosquitoes and are commonly found all over the island in urban, peri-urban and rural areas, sometimes reaching 1200–1400 m of altitude [37]. *Aedes aegypti* is also present on Reunion Island but its geographical distribution is restricted to a few local sites [37]. In 2005–2006, Reunion Island experienced a chikungunya virus (CHIKV) outbreak that was responsible for more than 250,000 human cases [38]. More recently, an epidemic of dengue virus (DENV) started at the end of 2017 was responsible for more than 25,000 autochthonous human cases on the island at the beginning of 2020 [39, 40]. Regarding ZIKV, although a small number of imported human cases have been reported, no autochthonous transmission has been recorded to date on Reunion Island. Previous investigations of vector competence for ZIKV involving mosquitoes from Reunion Island, only focused on *Ae. albopictus* and Asian ZIKV strains, reported no transmission capacity of the mosquito strains tested [41, 42].

In the present study, we examined the vector competence of three mosquito species from Reunion Island, namely *Ae. albopictus*, *Ae. aegypti* and *Cx. quinquefasciatus*, for one African and two Asian ZIKV strains. The results of this investigation may help to better estimate the risk of the ZIKV emergence on Reunion Island.

## Methods

### Mosquito samples

**Fig. 1:**
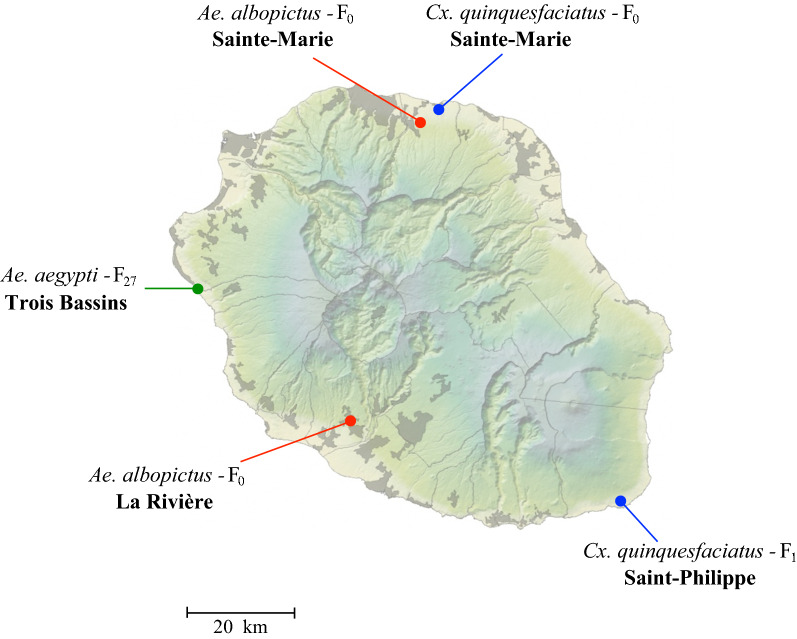
Map of sampling sites were mosquitoes were collected in Reunion Island. Red, green and blue points indicate the locations where *Aedes albopictus*, *Aedes aegypti* and *Culex quinquesfasciatus* strains were collected, respectively

Three mosquito species from Reunion Island were used to evaluate vector competence for ZIKV: *Ae. albopictus*; *Ae. aegypti*; and *Cx. quinquefasciatus*. Mosquitoes were sampled as eggs (for *Ae. albopictus*) or larvae and pupae (for *Cx. quinquefasciatus*) in four locations in 2019 (Fig. 1). Field samples were transported to the insect laboratory where they were reared to adulthood. For *Ae. aegypti*, a strain collected in 2014 (in the location Trois Bassins, see Fig. 1) and maintained in an insectary was used. Mosquitoes were reared under standard conditions at 26 ± 1 °C and 80% relative humidity (RH) with a 12 h light/12 h dark photoperiod. Larvae were supplied every two days with yeast tablets and adults were fed with 10% sucrose solution. Experimental infections were performed using 5 mosquito strains: 2 strains of *Ae. albopictus* (F_0_ generations); 1 strain of *Ae. aegypti* (F_27_ generation); and 2 strains of *Cx. quinquefasciatus* (F_0_ and F_1_ generations).

### Viral strains

We used three ZIKV strains, namely Dak84 (GenBank: KU955592, African lineage), MAS66 (GenBank: KX694533, Asian lineage) and PaRi_2015 (GenBank: KU647676, Asian lineage) provided as lyophilizates by the European Virus Archive goes global (EVAg). The Dak84 strain was originally isolated in 1984 from *Aedes taylori* in Senegal and passaged four times on BHK21 cells. The MAS66 strain was isolated in 1966 from *Ae. aegypti* from Malaysia and passaged four times on Vero cells. The PaRi_2015 strain was isolated from human serum in Martinique in 2015 and passaged three times on Vero cells. Before viral production, lyophilizates were re-suspended into 200 µl to 400 µl distilled water. All viruses were subsequently amplified in Vero E6 cells (ATCC, ref. CRL-1586) at a MOI of 0.1 in an Eagle’s minimum essential medium (MEM) supplemented with 2% heat-inactivated foetal bovine serum (FBS), 2 mmol/l l-glutamine, 1 mmol/l sodium pyruvate, 10 U/ml of penicillin, 0.1 mg/ml of streptomycin and 0.5 µg/ml of fungizone (PAN Biotech, Aidenbach, Germany). Vero cells were maintained at 37 °C with a 5% CO_2_ atmosphere. For all virus stocks, supernatants were harvested 2 days post-infection and then frozen at −80 °C until use. The final titers of our ZIKV stocks were 10^7.4^ PFU/ml, 10^5.8^ PFU/ml and 10^6.9^ PFU/ml for Dak84, MAS66 and PaRi_2015, respectively.

### Oral infections of mosquitoes

Seven to 10-day-old female mosquitoes were isolated in plastic boxes and transferred into a climatic chamber (26 ± 1 °C; 80% RH; 12 h light/12 h dark photoperiod) where they were starved for 48 h. After this starvation period, mosquitoes were fed with a blood meal containing 1.4 ml of washed rabbit erythrocytes, 700 μl of ZIKV containing culture supernatant from the strain of interest and 5 mM (21 μl) adenosine triphosphate (ATP) as phagostimulant. The titers of ZIKV in the blood preparations were 10^6.85^ PFU/ml, 10^5.3^ PFU/ml and 10^6.3^ PFU/ml for Dak84, MAS66 and PaRi_2015, respectively. The blood preparations containing ZIKV were provided to mosquitoes using a Hemotek feeding system (Hemotek Limited, Great Harwood, UK) with pig intestine as the membrane. After 30 min to 1 h of feeding, fully engorged females were transferred into a climatic chamber where they were maintained with 10% sucrose for 21 days.

### Mosquito processing

At 14 and 21 days post-infection (dpi), saliva was collected from individual mosquitoes (*n* = 10 to 33) using a forced salivation method [43] that allows saliva to be collected by removing the legs and wings of each mosquito and then inserting the proboscis into a 20 μl pipette tip containing 5 μl of FBS. After 45 min, the resulting solution containing the saliva was mixed with 45 μl of MEM supplemented with 2 mmol/l l-glutamine, 1 mmol/l sodium pyruvate, 10 U/ml of penicillin, 0.1 mg/ml of streptomycin and 0.5 µg/ml of fungizone. Afterwards, the head and the body of each specimen were ground separately in 300 μl of the same MEM medium but supplemented with 2% FBS and centrifuged at 10,000×*g* for 5 min to pellet tissue debris. Finally, 200 μl of the supernatant of each sample was stored at −80 °C until viral detection and titration.

### Virus detection, titration and vector competence evaluation

Plaque forming unit assays were used to determine the ZIKV infection status of bodies, heads and saliva. For this, Vero cells were seeded the previous day in 12-well culture plates at a density of 3 × 10^5^ cells per well. Cells were infected with 250 µl of ten-fold dilutions of body, head homogenates or saliva. After an incubation of 2 h at 37 °C with a 5% CO_2_ atmosphere, 1 ml of MEM supplemented with 5% of FBS, 2 mmol/l l-glutamine, 1 mmol/l sodium pyruvate, 10 U/ml of penicillin, 0.1 mg/ml of streptomycin, 0.5 µg/ml of fungizone and 0.8% carboxymethylcellulose sodium salt (CMC; Sigma-Aldrich, Saint-Quentin-Fallavier, France) was added in each well and cells were incubated for 7 days at 37 °C with a 5% CO_2_ atmosphere. Thereafter, the medium was removed, and cells were fixed with 3.7% paraformaldehyde (Sigma-Aldrich). Finally, cells were stained with 0.5% crystal violet (Sigma-Aldrich) diluted in 20% ethanol.

The vector competence of each mosquito strain for the three ZIKV strains was evaluated using samples collected only at 14 and 21 dpi as previous investigations have shown higher dissemination and transmission values from day 14 [17, 23, 44]. Three parameters were then examined: the infection rate (IR); the dissemination efficiency (DE); and the transmission efficiency (TE). The IR corresponds to the proportion of infected bodies (abdomen and thorax) among the total number of mosquitoes tested; the DE is the proportion of mosquitoes with ZIKV infected heads among all blood-fed females and the TE was calculated as the proportion of females with ZIKV in the saliva among the total number of mosquitoes tested.

### Statistical analysis

Fisher’s exact test was used to compare IR, DE and TE parameters for each dpi between mosquito strains for a given ZIKV strain. For multiple comparisons the Bonferroni correction was applied [45]. The Kruskal-Wallis test was used to compare viral loads in bodies, heads and saliva for each dpi between mosquito strains or species for a given ZIKV strain. All statistical analyses were performed in R software (v.3.6.2) [46] with the *FSA* [47], *RVAideMemoire* [48] and *stats* [46] packages.

## Results

Mosquito strains of *Ae. albopictus*, *Ae. aegypti* and *Cx. quinquesfasciatus* from Reunion Island were orally exposed to one African ZIKV strain (Dak84) and two Asian strains (PaRi_2015 and MAS66). We were unable to increase the titers of viral stocks by cell culture, therefore we fed each mosquito strain with the maximum possible virus titer, which differed between strains (i.e. 10^6.85^ PFU/ml, 10^5.3^ PFU/ml and 10^6.3^ PFU/ml for Dak84, MAS66 and PaRi_2015, respectively).

When infected with the ZIKV strain MAS66, no infectious ZIKV was detected in the bodies, the heads or the saliva of the two *Cx. quinquefasciatus* strains either at 14 or 21 dpi. Only one *Ae. albopictus* specimen out of 64 individuals from the location Sainte-Marie was able to be infected, to disseminate and to transmit MAS66 at 21 days with a viral titer of 6.48 log_10_ PFU, 6.35 log_10_ PFU and 1.52 log_10_ PFU detected in the body, the head and the saliva, respectively. The *Ae. aegypti* strain also showed a low susceptibility to MAS66 as infectious viral particles were only detected in the body of one specimen among 32 individuals at 14 dpi (viral load of 8.08 log_10_ PFU/body).

Vector competence analysis of the three mosquito species infected with the ZIKV strain PaRi_2015 showed no infection, dissemination or transmission for *Cx*. *quinquefasciatus* and *Ae. albopictus*. For *Ae. aegypti*, a limited number of specimens were able to replicate the ZIKV strain PaRi_2015. The values of IRs were 12.5% (*n* = 4) and 6.3% (*n* = 2) at 14 and 21 dpi, respectively (mean viral loads ± SD: 5.72 ± 0.36 log_10_ PFU/body and 5.59 ± 0.24 log_10_ PFU/body at 14 and 21 dpi, respectively); DEs of 9.4% (*n* = 3) and 6.3% (*n* = 2) at 14 and 21 dpi, respectively (mean viral loads: 4.73 ± 1.10 log_10_ PFU/head and 5.03 ± 0.15 log_10_ PFU/head at 14 and 21 dpi, respectively) and TEs of 3.1% (*n* = 1) at both 14 and 21 dpi (2.30 log_10_ PFU/saliva and 1.82 log_10_ PFU/saliva at 14 and 21 dpi, respectively).

**Fig. 2:**
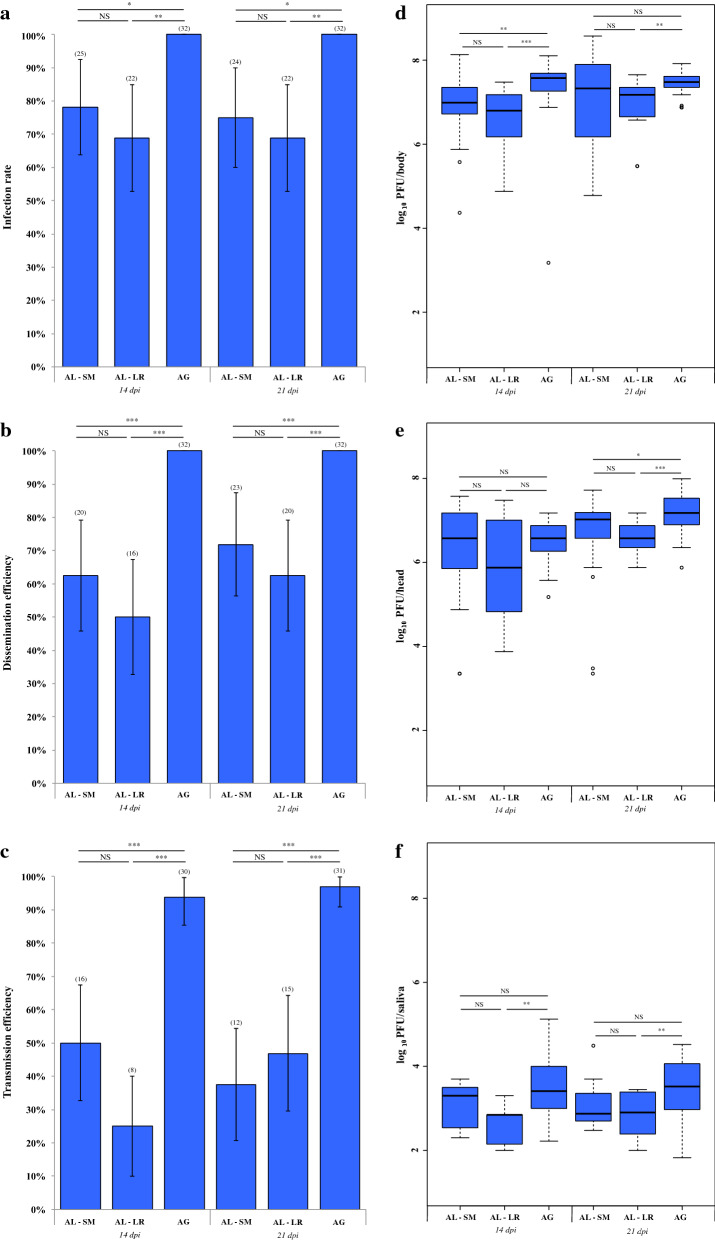
Vector competence parameters of *Aedes albopictus* and *Aedes aegypti* strains from Reunion Island infected with the African ZIKV strain Dak84 (infectious blood-meal viral titer of 10^6.85^ PFU/ml). At 14 and 21 days after the infection, mosquitoes were examined for the presence of infectious viral particles detected by titration on Vero cells. The graphics **a**–**c** correspond to infection rate (IR), dissemination efficiency (DE) and transmission efficiency (TE), respectively. The graphics **d**–**f** correspond to viral loads in bodies, heads and saliva, respectively. The numbers in parentheses indicate the number of positive samples (samples with infectious ZIKV particles). The error bars correspond to the 95% confidence interval. The asterisks indicate the level of significance of differences (NS: no significant difference; *P < 0.05; **P < 0.01; ***P < 0.001) obtained from Fisher’s exact tests (for infection rates, dissemination and transmission efficiencies) and Kruskal-Wallis tests (for viral loads in bodies, heads and saliva). *Abbreviations:* AG, *Ae. aegypti*; AL, *Ae. albopictus*; SM, Sainte-Marie; LR, La Rivière

The highest vector competence parameters were observed for the African ZIKV strain Dak84 from *Ae. albopictus* and *Ae. aegypti*, while *Cx. quinquefasciatus* mosquitoes were unable to be infected, to disseminate or to transmit Dak84 as observed with the two Asian ZIKV strains. For *Ae. albopictus*, the IRs were quite similar between the two mosquito strains at 14 dpi (78.1% and 68.8% for Sainte-Marie and La Rivière, respectively; Fig. 2a) and 21 dpi (75.0% and 68.8% for Sainte-Marie and La Rivière, respectively; Fig. 2a). Likewise, similar results were observed for DEs with 62.5% and 50.0% at 14 dpi and 71.9% and 62.5% at 21 dpi for Sainte-Marie and La Rivière strains, respectively (Fig. 2b). The comparison of the TEs between the two *Ae. albopictus* strains revealed lower values for mosquitoes from La Rivière at 14 dpi (25.0% for La Rivière *versus* 50.0% for Sainte-Marie), while the TE of La Rivière was higher than that of Sainte-Marie at 21 dpi (46.9% and 37.5% for La Rivière and Sainte-Marie, respectively). However, no significant differences were detected between the two *Ae. albopictus* strains for either TEs, IRs or DEs (Fisher’s exact test: all *P* > 0.05). Similarly, no significant differences were detected between the two mosquito strains for the viral loads in bodies (Kruskal-Wallis H-test: *χ*^2^ = 2.845, *df*  = 1, *P* = 0.092 at 14 dpi and *χ*^2^ = 0.779, *df*  = 1, *P* = 0.377 at 21 dpi), heads (Kruskal-Wallis H-test: *χ*^2^ = 1.053, *df*  = 1, *P* = 0.305 at 14 dpi and *χ*^2^ = 1.963, *df* = 1, *P* = 0.161 at 21 dpi) and saliva (Kruskal-Wallis H-test: *χ*^2^ = 4.603, *df* = 1, *P* = 0.032 at 14 dpi and *χ*^2^ = 1.108, *df*  = 1, *P* = 0.293 at 21 dpi) (Fig. 2d–f). For *Ae. aegypti*, all individuals were infected and were able to disseminate Dak84 at 14 and 21 dpi (IRs = 100.0% and DEs = 100.0%) (Fig. 2a, b). In addition, almost all individuals were able to transmit Dak84 (TEs of 93.8% and 96.9% at 14 and 21 dpi, respectively) (Fig. 2c). For this African ZIKV strain, in bodies, the mean viral loads were 7.36 ± 0.81 log_10_ PFU/body and 7.41 ± 0.30 log_10_ PFU/body at 14 and 21 dpi, respectively. In heads, the mean viral loads were 6.51 ± 0.51 log_10_ PFU/head and 6.61 ± 0.35 log_10_ PFU/head at 14 and 21 dpi, respectively. In saliva, the mean viral loads were 3.47 ± 0.72 log_10_ PFU/saliva and 3.45 ± 0.70 log_10_ PFU/saliva at 14 and 21 dpi, respectively (Fig. 2d–f).

When comparing vector competence parameters of *Ae. aegypti* and *Ae. albopictus* infected with Dak84, the IRs, DEs and TEs were significantly higher in *Ae. aegypti* than in *Ae. albopictus* (Fisher’s exact test: all; *P* < 0.05 for IRs, DEs and TEs at 14 and 21 dpi) (Fig. 2a–c). Similarly, *Ae. aegypti* displayed significantly higher viral loads in bodies at 14 dpi (Kruskal-Wallis H-test: *χ*^2^ = 22.315, *df* = 1, *P* < 0.001) and 21 dpi (Kruskal-Wallis H-test: *χ*^2^ = 6.933, *df* = 1, *P* = 0.008 at 21 dpi), in heads at 21 dpi (Kruskal-Wallis H-test: *χ*^2^ = 14.897, *df* = 1, *P* < 0.001), and in saliva at 14 dpi (Kruskal-Wallis H-test: *χ*^2^ = 6.098, *df*  = 1, *P* = 0.014) and 21 dpi (Kruskal-Wallis H-test*: χ*^2^ = 8.512, *df* = 1, *P* = 0.004 at 21 dpi) (Fig. 2d–f). However, no significant difference in viral loads in heads at 14 dpi was detected between the two mosquito species (Kruskal-Wallis H-test: *χ*^2^ = 0.382 *df* =  = 1, *P* = 0.537).

## Discussion

In the present study, we evaluated the vector competence of three mosquito species from Reunion Island including *Ae. albopictus*, *Ae. aegypti* and *Cx. quinquefasciatus* for three ZIKV strains: one African strain (Dak84) and two Asian strains (PaRi_2015 and MAS66). We showed that *Cx. quinquefasciatus* mosquitoes were unable to be infected, to disseminate or to transmit the three ZIKV strains, a result also reported by Hery et al. [23] using *Cx. quinquefasciatus* mosquitoes from Guadeloupe tested against the same three ZIKV strains. Our results are also consistent with other studies showing that *Cx. quinquefasciatus* from different geographical origins, as well as its sibling species *Cx. pipiens*, are not able to transmit ZIKV [19–29]. This refractoriness against ZIKV does not seem to be related to an RNA interference (RNAi) mechanism or to the presence of the endosymbiotic bacterium *Wolbachia* [20]. However, it could be explained by other antiviral immune responses in the midgut that block virus replication [49] or the absence of specific cell receptors for attachment of ZIKV on midgut epithelial cells [50]. Ultimately, our results suggest that *Cx. quinquefasciatus* would not be a vector of ZIKV on Reunion Island.

The *Ae. albopictus* and *Ae. aegypti* strains from Reunion Island were poorly competent for the two Asian ZIKV strains at the viral titers tested. Indeed, for *Ae. albopictus*, no infectious viral particle was detected with the PaRi_2015 strain and only one specimen was able to transmit the MAS66 strain. In contrast, mosquitoes from the two tested *Ae. albopictus* strains were more susceptible to the African ZIKV Dak84 strain (IRs, DEs and TEs up to 78.1%, 62.5% and 50.0%, respectively). The results obtained for *Ae. aegypti* were quite similar to those of *Ae. albopictus* with no transmission for MAS66, only two positive saliva for PaRi_2015, and higher transmission efficiencies with Dak84 (TEs of 93.8% and 96.9% at 14 and 21 dpi, respectively). Such a difference in vector competence of *Ae. albopictus* and *Ae. aegypti* between Asian and African ZIKV strains could be explained by virus titers used in infectious blood meals. Indeed, lower titers were used for the two Asian ZIKV strains (10^5.3^ PFU/ml and 10^6.3^ PFU/ml, respectively for MAS66 and PaRi_2015) as compared to Dak84 with 10^6.85^ PFU/ml; and the effects of blood-meal titers in the outcomes of vector competence analyses involving ZIKV strains have been previously demonstrated [51, 52]. Despite this caveat, several studies also described low transmission rates of Asian ZIKV strains by *Aedes* mosquitoes, even using higher virus titers. For Reunion *Ae. albopictus*, no viral transmission was detected using Asian ZIKV strains from New Caledonia (NC-2014-5132, virus titer of 10^7^ TCDI_50_/ml) [41] or from Suriname (SL1602, virus titers of 7.5 × 10^6^ FFU/ml and 3 × 10^6^ FFU/ml) [42]. On the contrary, other studies have reported relatively high transmission efficiencies of Asian ZIKV strains using mosquito strains with different geographical origins. For instance, vector competence experiments have demonstrated the transmission of the ZIKV strains NC-2014-5132 and SL1602 by *Ae. albopictus* strains from Africa, America and France [31, 44, 53]. For *Ae. aegypti,* low susceptibility to Asian ZIKV strains has also been reported [17, 32, 44] whereas higher transmission efficiencies were achieved from an *Ae. aegypti* strain from Guadeloupe (TEs of 12% for infection with MAS66 and PaRi_2015) [23] and from French Polynesia (TE of 17% for the ZIKV strain NC-2014-5132) [17]. Taken together, this supports the idea that vector competence of *Aedes* mosquitoes is population- and ZIKV strain-dependent. Some mosquito strains are competent to transmit ZIKV while others are poorly competent or cannot transmit the virus [17, 23, 26, 31–33, 41, 44, 54–59].

The role of mosquito-ZIKV interaction in the outcome of vector competence is also illustrated by the African ZIKV strain. Indeed, when comparing our results with those of Hery et al. [23] we obtained higher TEs from an equivalent titer of the Dak84 strain for *Ae. aegypti* mosquitoes from Reunion (mean TE > 90%) than reported for *Ae. aegypti* from Guadeloupe (mean TE = 50%). Differences were also observed with viral loads in saliva with higher viral concentrations in mosquitoes from Reunion Island (mean viral load of 3.47 ± 0.72 log_10_ PFU/saliva and 3.45 ± 0.70 log_10_ PFU/saliva at 14 and 21 dpi, respectively) as compared to those from Guadeloupe (mean viral concentration inferior to 2.0 log_10_ PFU/saliva) [23]. Such contrasting results between the two studies could be explained by the laboratory colonization, with a laboratory *Ae. aegypti* strain of several generations (F_27_ generation) for our study *versus* a F_1_ mosquito strain for Hery et al. [23]. Other factors affecting the outcomes of vector competence such as the temperature [60] and feeding conditions [34] could also explain these differences.

Finally, although we used different virus titers for Asian and African ZIKV strains, both Reunion *Ae. albopictus* and *Ae. aegypti* appeared more able to transmit the African ZIKV strain. This result is consistent with previous investigations showing that even with similar titers of ZIKV strains in infectious blood meals, African ZIKV strains are more efficiently transmitted by mosquitoes than Asian strains [18, 32–34, 36].

## Conclusions

Our results showed that *Cx. quinquefasciatus* from Reunion Island is refractory to ZIKV infection. In contrast, *Ae. albopictus* and *Ae. aegypti* from Reunion Island showed low vector competence for Asian ZIKV strains and displayed high transmission efficiencies for the African ZIKV. Moreover, we found that *Ae. aegypti* was a modestly more efficient vector for the African ZIKV strain than *Ae. albopictus* which is consistent with a recent study conducted in Central Africa with the Dak84 ZIKV strain [61]. However, on Reunion Island, *Ae. albopictus* is far more abundant than *Ae. aegypti* and therefore may play a more significant role in ZIKV transmission even in light of the differential vector competence. Future studies should also consider potential mutations in the viral genome that can change the vector competence, as was reported for CHIKV where a single substitution was associated with better dissemination by *Ae. albopictus* [62, 63].

## Data Availability

All data generated or analysed during this study are included in this published article.

## Abbreviations

CHIK: Chikungunya virus
DE: Dissemination efficiency
DENV: Dengue virus
Dpi: Days post-infection
MEM: Eagle’s minimum essential medium
FBS: Foetal bovine serum
IR: Infection rate
RH: Relative humidity
TE: Transmission efficiency
ZIKV: Zika virus
